# Role of Fluxes in Optimizing the Optical Properties of Sr_0.95_Si_2_O_2_N_2_:0.05Eu^2+^ Green-Emitting Phosphor

**DOI:** 10.3390/ma6072862

**Published:** 2013-07-15

**Authors:** Lihong Liu, Rong-Jun Xie, Chenning Zhang, Naoto Hirosaki

**Affiliations:** 1 Structural and Functional Integration of Ceramics Group, the Division of Functional Materials and Nanodevices, Ningbo Institute of Materials Technology and Engineering, Chinese Academy of Sciences, Ningbo, Zhejiang 315201, China; E-Mail: liu.lihong@nims.go.jp; 2 Sialon Group, Sialon Unit, National Institute for Materials Science, Namiki 1-1, Tsukuba, Ibaraki 305-0044, Japan; E-Mail: hirosaki.naoto@nims.go.jp; 3 Fine Particles Engineering Group, Materials Processing Unit, National Institute for Materials Science, Tsukuba, Ibaraki 305-0047, Japan; E-Mail: zhang.chenning@nims.go.jp

**Keywords:** photoluminescence properties, oxynitride phosphor, flux, green emitting

## Abstract

Chlorides of NH_4_Cl and SrCl_2_ and fluorides of AlF_3_ and SrF_2_ were added to raw materials acting as the flux for preparing the SrSi_2_O_2_N_2_:Eu^2+^ phosphor. The effects of the fluxes on the phase formation, particle morphology, particle size, and photoluminescence properties were investigated. The results revealed that particle size, particle morphology and photoluminescence intensity were largely dominated by the type of the flux material and its adding amount. The chloride-based flux was found to favor the formation of the SrSi_2_O_2_N_2_:Eu^2+^ phase. Among the chloride-based fluxes, the sample added with the SrCl_2_ flux presented the narrow particle distribution and cleaner surface, with enhanced emission intensity and an increased external quantum efficiency by 68% and 22%, respectively, compared with those of the sample without any flux adding.

## 1. Introduction

Recently, phosphor converted white light-emitting diodes (LEDs) have attracted much attention due to their excellent properties of high efficiency, long lifetime, low power consumption and environment-friendly characteristic, compared with those of conventional incandescent and fluorescent lamps [1,2,3,4,5,6,7,8,9,10]. Normally, there are two methods to generate white light for phosphor-converted white LEDs. The first method is combination of the blue LED chip with yellow phosphor; the second method is combination of the UV LED chip with blue, green, and red phosphors [3]. As the phosphor is one of the important parts in white LEDs, the photoluminescence properties of the phosphor can determine the performance of the LED device. In order to successfully apply the phosphor-combined LED to the lighting, the used phosphor must work under long-wavelength UV or blue LED chips and have high emission efficiency. There are some factors to affect the optical properties of the phosphor, such as particle size, crystallinity, particle morphology, and composition homogeneity. In addition, defects in the crystal grain are also a main factor to affect the luminescence of phosphor. To overcome the drawbacks mentioned above, adding flux in raw material in the solid-state reaction among various synthetic processes have been used [11,12,13,14,15,16]. For the flux used, a flux material has a melting point below the solid-state reaction temperature, which can dissolve one or more reaction components and allow the materials to enter the reaction zone. Therefore, the flux addition has great influence on the ion diffusions in the solid-state reaction and particle size distribution, growth condition, crystallization process of the synthesized phosphor as well as the formation of target product matrix with good crystallinity. Besides, by using the flux material, one of the big advantages to be taken is known to reduce the processing temperature, leading to lower the production cost. Previous literature [17,18,19] has reported that fluxes adding into various phosphors could improve the morphological and optical properties of the phosphor powders. Although flux materials, in general, can optimize the optical properties of the phosphors, the selection of an appropriate flux material and understanding of flux effect on the optical properties may differ from one phosphor to another. 

In this work, Sr_1–*x*_Si_2_O_2_N_2_:*x*Eu^2+^ phosphor is taken as a sample to understand the role of flux. This phosphor is a new class of green-emitting phosphors used into the white light-emitting diodes (WLEDs), which have superior properties over sulfide and oxide phosphors. The purpose of this work is to add various fluxes (NH_4_Cl, SrCl_2_, AlF_3_, SrF_2_) into the raw materials to investigate the effect of flux on the photoluminescence properties and crystal formation of the sample so that an appropriate flux for synthesizing Sr_1–*x*_Si_2_O_2_N_2_:*x*Eu^2+^ would be selected. We concentrate on controlling the particle size, particle morphology, and photoluminescence intensity by combining a firing temperature with an appropriate flux, which is an important factor from the applicability of view point.

## 2. Results and Discussion

**Figure 1:**
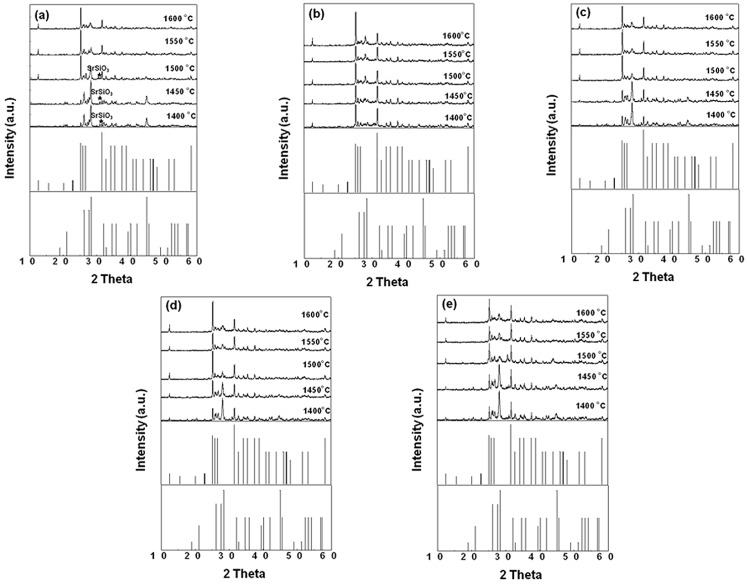
XRD patterns of SrSi_2_O_2_N_2_:Eu^2+^ sintered at various temperatures with 1% flux (**a**) NH_4_Cl; (**b**) AlF_3_; (**c**) SrCl_2_; (**d**) SrF_2_; (**e**) none.

Figure 1 gives the XRD patterns of SrSi_2_O_2_N_2_:Eu^2+^ with 1 wt % adding amount of different fluxes (NH_4_Cl, SrCl_2_, SrF_2_ and AlF_3_) firing at various temperatures. It can be seen from Figure 1a that when the sample doped with NH_4_Cl flux was sintered below 1550 °C, the resultant powder was composed of a main phase of SrSi_2_O_2_N_2_ (JCPD card No. 49−0839) and a few SrSiO_3_ (JCPD No. 36−0018) impurity phase. Further increased the sintering temperature to 1550 °C, the XRD diffraction peaks matched well with those in SrSi_2_O_2_N_2_ structure with JCPD card No. 49–0840. This indicated that the starting phosphor materials were prone to the low temperature type of SrSi_2_O_2_N_2_ when the sintered temperature was below 1550 °C, whereas the high temperature type of SrSi_2_O_2_N_2_ was attained at a sintered temperature of above 1550 °C. This result is consistent with that reported in the literature [20]. This lower phase transformation temperature may be due to that NH_4_Cl has a decomposition reaction at a much lower temperature than that for the sample synthesis and could be flushed out of the system by the gas flow. As a result, the effect of NH_4_Cl as the flux in this reaction may play a role in cleaning effect for the surfaces of the starting particles to improve the reaction reactivity and therefore lower the synthesis temperature of the samples. For the starting materials doped with AlF_3_, SrCl_2_, SrF_2_ fluxes and without any flux, the high temperature phase of SrSi_2_O_2_N_2_ was obtained at sintering temperatures above 1400, 1500, 1500, and 1550 °C, respectively. The results above show that adding the fluxes into the raw materials can reduce the phase transformation temperature of SrSi_2_O_2_N_2_. Moreover, there is not any appreciable flux diffraction peaks could be found in the XRD patterns, indicating that ignorable effect of the flux addition on unpurifying the resultant phosphors. 

**Figure 2:**
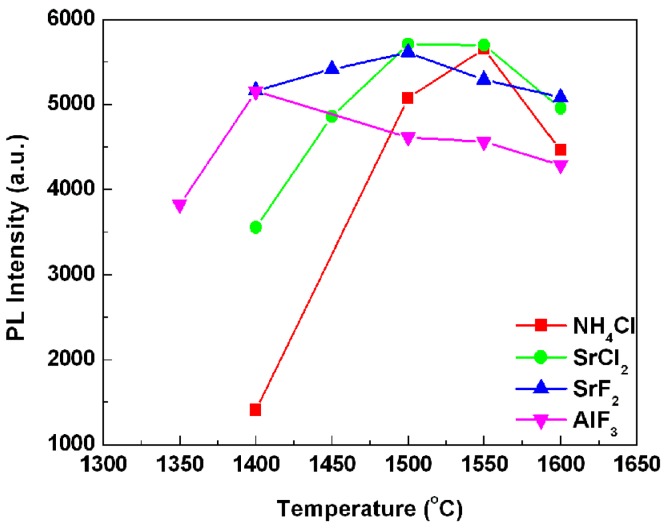
Emission intensities of the SrSi_2_O_2_N_2_:Eu^2+^ sintered at various temperatures with 1% flux.

Figure 2 presents the sintering temperature dependent emission intensity of the phosphors synthesized above. Note that, with the increase of the sintering temperature, the emission intensities of all the samples increased firstly, and then fell down after the emission intensity reached the maximum value. The optimal synthesis temperatures of these samples were mainly dependent on the flux types. For the phosphors with added NH_4_Cl, AlF_3_, SrCl_2_, and SrF_2_ fluxes, the optimal sintering temperatures were 1550, 1400, 1500, and 1500 °C, respectively, which are corresponding to the lowest phase transformation temperatures of SrSi_2_O_2_N_2_, as shown in the XRD patterns (Figure 1), indicating that the emission intensity of phosphor composed of SrSi_2_O_2_N_2_ high temperature phase was much higher than that of the sample with SrSi_2_O_2_N_2_ low temperature phase. Thus it can be seen that adding flux can reduce the optimal sintered temperature and enhance the crystallinity effectively, since fluxes melt at lower temperature than the solid-state reaction temperature and act as solvents for the reactants facilitating solid-state reaction, finally resulting in the enhanced emission intensity at a lower sintering temperature.

**Figure 3:**
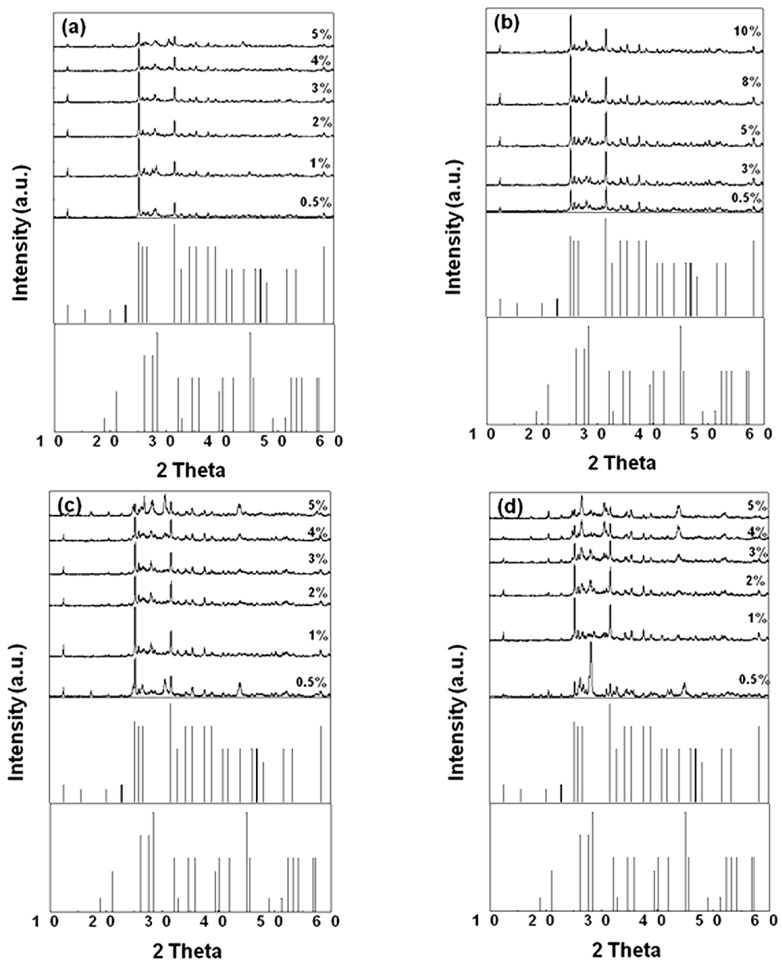
XRD patterns of SrSi_2_O_2_N_2_:Eu^2+^ adding various amounts flux (**a**) NH_4_Cl (1550 °C); (**b**) SrCl_2_ (1500 °C); (**c**) SrF_2_ (1500 °C); (**d**) AlF_3_ (1400 °C).

To further investigate the effects of flux on the phase formation and photoluminescence properties of SrSi_2_O_2_N_2_, we have synthesized SrSi_2_O_2_N_2_ samples with various adding amounts of different fluxes. The XRD patterns of SrSi_2_O_2_N_2_ doped with various amounts of the fluxes are shown in Figure 3. It can be seen that, for the sample doped with NH_4_Cl flux, when the NH_4_Cl adding amount was increased to 5%, SrSiO_3_ impurity phase was observed in the resultant sample. The presence of the impurity phase was also observed in the samples doped with certain adding amounts of SrF_2_ and AlF_3_ fluxes. However, for the samples doped with various adding amounts of SrCl_2_ flux, all the resultant powders were composed of a single high temperature phase of SrSi_2_O_2_N_2_. Therefore, it can be concluded that appropriate flux adding amount is very important for obtaining the SrSi_2_O_2_N_2_ with single high temperature phase.

**Figure 4:**
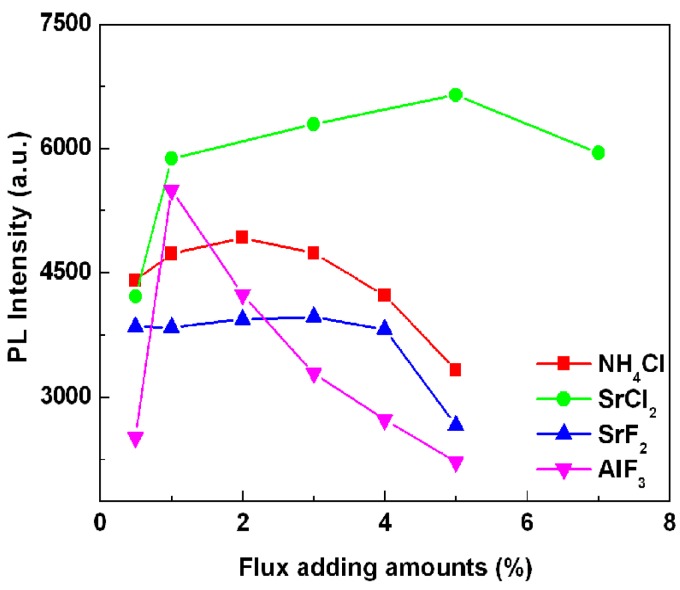
Effects of flux adding amounts on the emission intensity of SrSi_2_O_2_N_2_:Eu^2+^ phosphor with various kinds of flux.

Figure 4 gives the effect of the addition amount of various types of fluxes on the emission intensity of SrSi_2_O_2_N_2_:Eu^2+^ phosphor. Note that, the emission intensity enhanced with the increase of the flux adding amount, the strongest emission intensities were obtained for the samples added with 2 wt %, 5 wt %, 3 wt % and 1 wt % of NH_4_Cl, SrCl_2_, SrF_2_ and AlF_3_ fluxes. Then the emission intensity was decreased as the adding amounts were further increased. Furthermore, it can be seen from Figure 3 that the strongest emission intensity was achieved for the samples composed of SrSi_2_O_2_N_2_:Eu^2+^ high temperature phase, which indicates that SrSi_2_O_2_N_2_:Eu^2+^ high temperature phase is advantage to obtain stronger photoluminescence properties. From Figure 4, it is known that the proper flux adding amount used in synthesizing sample can obtain optimal emission intensity of the phosphor. This may be due to that the added flux at proper flux adding amount is favorable for controlling the particle size distribution, particle morphology and the crystallinity of the resultant powders. 

**Figure 5:**
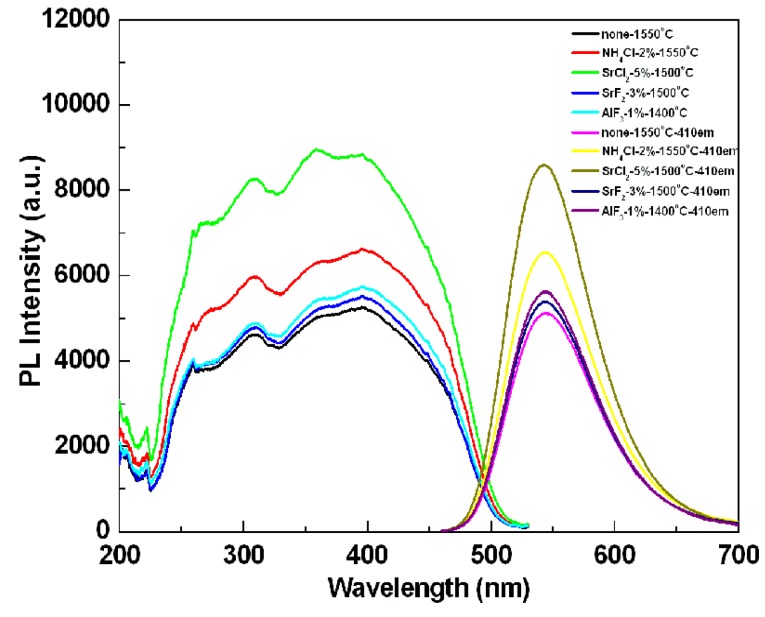
Effects of various kinds of flux on the emission intensity of SrSi_2_O_2_N_2_:Eu^2+^ phosphors.

**Figure 6:**
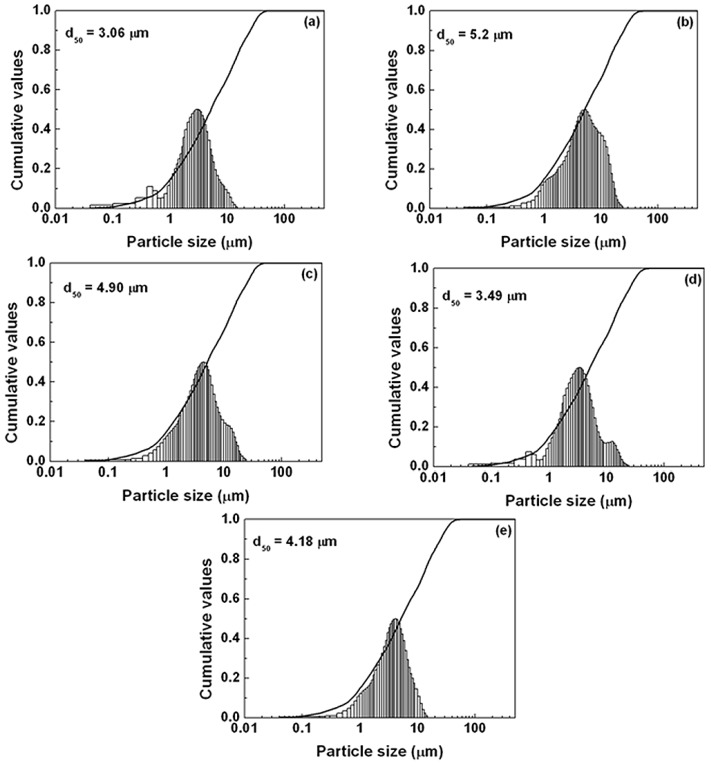
Particle size distribution of SrSi_2_O_2_N_2_:Eu^2+^ with various kinds of flux (**a**) none (1550 °C); (**b**) NH_4_Cl (1550 °C, 2%); (**c**) SrCl_2_ (1500 °C, 5%); (**d**) SrF_2_ (1500 °C, 3%); (**e**) AlF_3_ (1400 °C, 1%).

**Figure 7:**
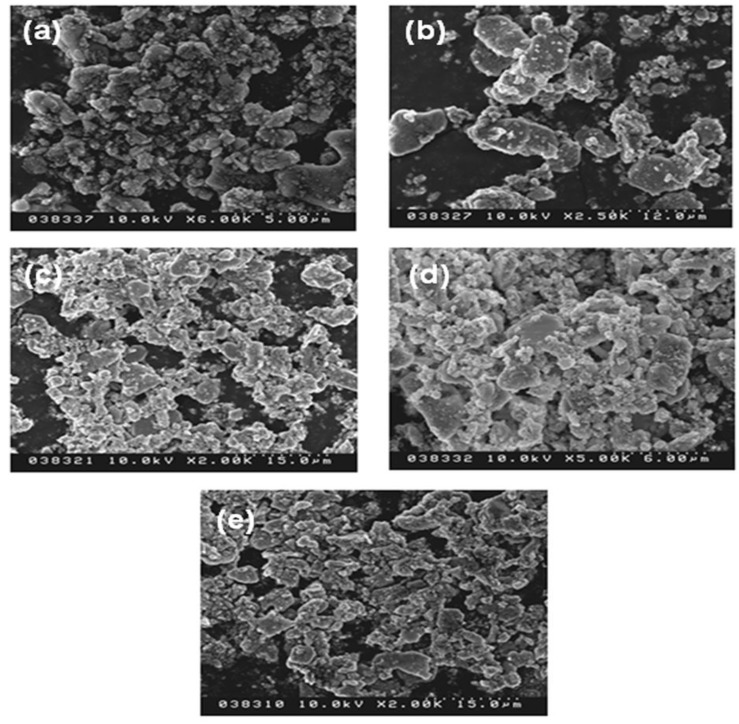
SEM images of SrSi_2_O_2_N_2_:Eu^2+^ with various kinds of flux (**a**) none (1550 °C); (**b**) NH_4_Cl (1550 °C, 2%); (**c**) SrCl_2_ (1500 °C, 5%); (**d**) SrF_2_ (1500 °C, 3%); (**e**) AlF_3_ (1400 °C, 1%).

To compare the photoluminescence properties of the SrSi_2_O_2_N_2_:Eu^2+^ phosphors added with and without the flux, the photoluminescence spectra have been determined, as shown in Figure 5. The emission intensity of the sample after adding the flux was stronger than that of the powder without any flux. The reason for increasing emission intensity by adding flux should be ascribed to the crystallinity improvement of the synthesized particles, which would reduce the defects in the lattice and on the surface of the phosphor; simultaneously, and the growth enhancement of the particle size and reduction of the size distribution, which would decrease the light scattering caused by the small particles. The particle size of the synthesized sample without any flux was about 3 µm, whereas the particle sizes of the samples added with NH_4_Cl, SrCl_2_, SrF_2_ and AlF_3_ were 5.2, 4.9, 3.49 and 4.18 µm, respectively (see Figure 6). Additionally, another reason responsible for the increased emission is the control of the particle morphology by adding the flux. As seen Figure 7, the sample without adding any flux had small particle size and the particle was not crystallized very well; in contrast, the sample with adding flux had uniform particle morphology, which is advantage to obtain excellent photoluminescence properties. For the samples using various fluxes, the sample added with SrCl_2_ had the strongest emission intensity. Compared with the samples added without any flux and with the fluxes of NH_4_Cl, AlF_3_ and SrF_2_, the emission intensity of the SrSi_2_O_2_N_2_:Eu^2+^ with SrCl_2_ addition was enhanced evidently. This may be ascribed to every given host material having its proper flux material, which enhances the crystallinity and particle morphology of the resultant powder, finally resulting in improved photoluminescence properties. In our work, the emission intensity was much higher for the sample added with the chlorid fluxes than the sample added with the fluorid fluxes. This may be due to that the melting point of the chlorid flux was much lower than that of the fluorid flux, making the chloride liquid form easily before the formation of the low temperature phase of the SrSi_2_O_2_N_2_:Eu^2+^, which would enhance the distribution of the Eu^2+^ ions in the crystal structure. Furthermore, the existence of the liquid phase could increase the contact of the raw material particles and enhance the diffusions of the materials, consequently improving the reaction activity of the powders. For the sample with adding SrCl_2_ flux has the strongest emission intensity, which may be considered that the Sr^2+^ ions can compensate the losing Sr^2+^, which was caused by the volatilization of the released Sr from the SrO decomposition during the reaction.

**Figure 8:**
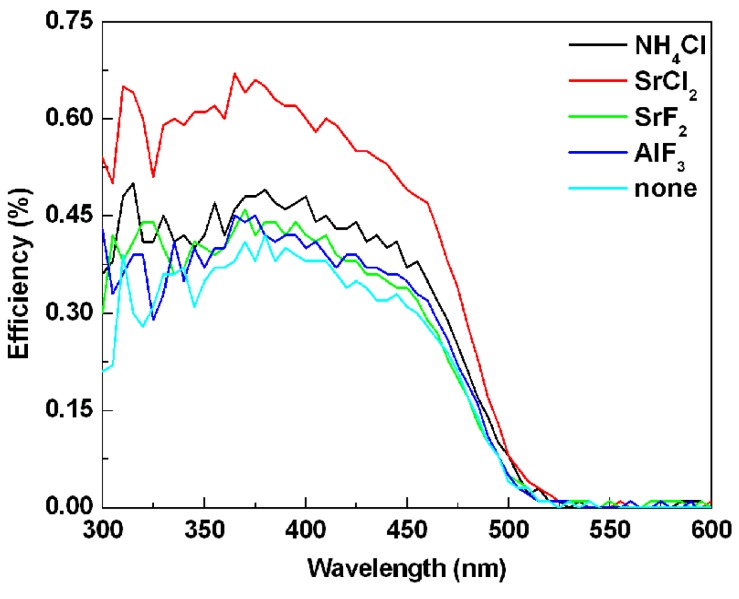
Effects of various kinds of flux on the external quantum efficiencies of SrSi_2_O_2_N_2_:Eu^2+^ phosphors.

Figure 8 gives the external quantum efficiencies of the SrSi_2_O_2_N_2_:Eu^2+^ doped with and without fluxes. Because of the apparatus calibration and stability of determined oxynitride powder in ambient, the degree of confidence on efficiency data can be considered to be qualified. It can be seen that the quantum efficiencies of all the samples doped with flux were stronger than that of the sample without any flux. The external quantum efficiencies of the samples doped with NH_4_Cl, SrCl_2_, SrF_2_ and AlF_3_ were 45%, 60%, 42% and 39%, respectively, under 410 nm excitation. Whereas, the external quantum efficiency of sample doped without any flux was 38%. The enhanced quantum efficiencies may be due to that the crystallinity of the powders was enhanced by adding the flux, resulting in the reduction of the defects in the host lattice and on the surface of the particles. As a result, the possibility of the non-radiation transition within and among the particles was reduced. 

## 3. Experimental Procedures

A series of the Sr_1−*x*_Si_2_O_2_N_2_:*x*Eu^2+^ (Eu^2+^ = 5 mol %) samples added with the fluxes of 0.5%–5% of NH_4_Cl, SrF_2_, AlF_3_ and 0.5%–7% of SrCl_2_ were synthesized by the solid-state reaction technique at high temperature. Stoichiometric mixtures of SrCO_3_ (Kojundo Chemical Laboratory Co. Ltd., Tokyo, Japan), SiO_2_ (Kojundo Chemical Laboratory Co. Ltd., Japan), Eu_2_O_3_ (Shin-Etsu Chemical Co. Ltd., Tokyo, Japan), Si_3_N_4_ (SN-E10, Ube Industries, Tokyo, Japan), NH_4_Cl (Sigma-Aldrich K.K., Tokyo, Japan), SrCl_2_ (Wako pure chemical industries Ltd., Louis, USA), SrF_2_ (Sigma-Aldrich K.K., Tokyo, Japan), and AlF_3_ (Kojundo Chemical Laboratory Co. Ltd., Tokyo, Japan) were weighed out and grounded. The mixed powders were then fired in N_2_/5% H_2_ between 1400 °C and 1600 °C for 6 h in a tube furnace. After the firing, the power was shut off and the sample was cooled down with the furnace. 

The phase purity was determined by X-ray powder diffraction (XRD) (Rigaku, RINT Ultima-III) with Cu-K_α_ radiation (λ = 1.54056 Å), operating at 40 kV and 40 mA. The XRD data were collected in the range of 10°–60° in 2*θ* by a step-scan mode with a step size of 0.02. 

Photoluminescence spectra were measured at room temperature by a fluorescent spectrophotometer (F-4500, Hitachi Ltd., Tokyo, Japan) with a 200 W Xe-lamp as an excitation source. Emission spectra were corrected for the spectral response of a monochromater and Hamamatsu R928P photomultiplier tube by a light diffuser and tungsten lamp (Noma, 10 V, 4 A). Excitation spectra were also corrected for the spectral distribution of xenon lamp intensity by measuring Rhodamine-B as a reference. The quantum efficiency was determined on a Hamamatsu MPCD-7000 multichannel photo detector with a 200 W Xe-lamp as an excitation source. 

The powder morphology was investigated by field-emission scanning electron microscopy (FESEM, JEOL-840A, Tokyo, Japan). The particle size distribution was recorded on the Microtrac particle size analyzer (CILAS 1064, Wisconsin, USA).

## 4. Conclusions 

In order to enhance the photoluminescence properties of SrSi_2_O_2_N_2_:Eu^2+^ produced by a conventional solid state reaction, a variety of fluxes were used and their effects on photoluminescence were investigated. It was found that the flux material played a key role in synthesizing the SrSi_2_O_2_N_2_:Eu^2+^ phosphor particles via the solid state reaction. The particle size, particle morphology and photoluminescence intensity were largely dependent on the type of the flux material used and its addition amount. The melting point of the flux materials relative to the synthesizing temperature of the reaction was found to be an important consideration for selecting an appropriate flux. The chloride-based flux was found to work well for the formation of the SrSi_2_O_2_N_2_:Eu^2+^ phase. Among the chloride-based flux, the sample added with SrCl_2_ had a narrow particle distribution and clean surface. The emission intensity and external quantum efficiency of the SrSi_2_O_2_N_2_:Eu^2+^ phosphor added with SrCl_2_ was enhanced by 68% and 22%, respectively, compared with those obtained from the sample without any flux.
